# Overexpression of a Multiprotein Bridging Factor 1 Gene *DgMBF1* Improves the Salinity Tolerance of Chrysanthemum

**DOI:** 10.3390/ijms20102453

**Published:** 2019-05-17

**Authors:** Qian Zhao, Ling He, Bei Wang, Qinglin Liu, Yuanzhi Pan, Fan Zhang, Beibei Jiang, Lei Zhang

**Affiliations:** Department of Ornamental Horticulture, Sichuan Agricultural University, 211 Huimin Road, Wenjiang District, Chengdu 611130, China; s20167109@stu.sicau.edu.cn (Q.Z.); heling@stu.sicau.edu.cn (L.H.); s20167108@stu.sicau.edu.cn (B.W.); scpyzls@sicau.edu.cn (Y.P.); 13305@sicau.edu.cn (F.Z.); 13786@sicau.edu.cn (B.J.); 14069@sicau.edu.cn (L.Z.)

**Keywords:** multiprotein bridging factor 1, *DgMBF1*, transgenic chrysanthemum, salt stress tolerance, gene expression

## Abstract

Soil salinity represents a major constraint in the growth of chrysanthemum. Therefore, improving salinity tolerance of chrysanthemum has become an important research direction in tolerance breeding. Multiprotein bridging factor 1 (MBF1) is an evolutionarily highly conserved transcriptional co-activator in archaea and eukaryotes and has been reported to play important roles to respond to abiotic stresses. Here, a MBF1 gene induced by salt stress was isolated and functionally characterized from *Dendranthema grandiflorum* and name as *DgMBF1*. Overexpression of *DgMBF1* in chrysanthemum increased the tolerance of plants to high salt stress compared to wild type (WT). It also showed fewer accumulations of hydrogen peroxide (H_2_O_2_), superoxide anion (O_2_^−^), higher activities of antioxidant enzymes, more content of proline and soluble sugar (SS) and more favorable K^+^/Na^+^ ratio than those of WT under salt stress. In addition, the expression level of genes related to antioxidant biosynthesis, proline biosynthesis, glyco-metabolism and K^+^/Na^+^ homeostasis was statistically significant higher in the *DgMBF1*-overexpressed lines than that in WT. These results demonstrated that *DgMBF1* is a positive regulator in response to salt stress and could serve as a new candidate gene for salt-tolerant plant breeding.

## 1. Introduction

Land salinization is a worldwide ecological issue. As one of the limiting factors, it severely hampers the growth of plants and the production of crops [1,2]. It has been determined that plants can effectively respond to environmental stress by identifying a series of complex biological signals and activating its transduction mechanism [3]. Generally, two phases of stress are revealed after the occurrence of salt stress: a rapid osmotic stress and a slower ionic stress [4]. Accordingly, there are three major physiological adaptive mechanisms of salt tolerance: osmotic stress tolerance, maintenance of ions homeostasis, and compartmentalization of Na^+^ to reduce cytosolic Na^+^ concentrations [5]. In addition, reactive oxygen species (ROS) plays a key role in the response to salt stress. ROS is a kind of toxic molecules that causes oxidative damage to proteins, DNA and lipids [6]. Under normal conditions, ROS was produced at a low level in organelles such as chloroplasts, mitochondria and peroxisomes. Under salt stress, the production rate of ROS is dramatically elevated. The balance between production and elimination of ROS determines its accumulation greatly [7].

At present, billions of hectares of various saline-alkali land exist globally, accounting for one-tenth of the world’s arable land. Twenty-three percent of cultivated land is salt-affected. [8] Chrysanthemums are produced mainly by facility cultivation. Due to the continuous increase of irrigation times in the facility, the transpiration of chrysanthemum speeds. However, the rainwater cannot take the salt away in time. Deep in the soil layer, the salt remains completed [9]. The continuous planting makes the salt accumulate year by year in soil. It poses a serious impact on the production and quality of chrysanthemums. Therefore, our question is how to cultivate and breed chrysanthemums under strong salt tolerance became a botanical concern. Genetic engineering methods can be used to transfer salt-tolerant genes into plants, which significantly improve the salt tolerance in transgenic plants.

Transcriptional regulatory proteins were involved in a variety of biological processes, especially in the expression of genomic information [10,11,12]. Among these proteins, transcriptional co-activators interacted with transcription factors, regulatory elements and the basal transcription machinery to complete the eukaryotic gene expression [13]. MBF1 proteins functioned as a non-DNA binding transcriptional co-activator. By bridging TATA box binding proteins, specific transcription factors were activated. The transcription of its target genes was enhanced to bind to target promoters in eukaryotes and to participate in multiple growth and developmental processes [14,15]. 

MBF1s elevated the regulation of multiple biological processes in yeast and animals [16,17]. MBF1s were also known to participate in a variety of abiotic and biotic stresses responses in plants [18]. There were three different homologs that could encode MBF1 in *Arabidopsis thaliana*. Their existence complemented the deletion of MBF1 in yeast [19]. The expression of *AtMBF1a* and *AtMBF1b* were regulated by plant development. The overexpression of *AtMBF1a* improved salt tolerance, fungal resistance and glucose insensitivity in transgenic *Arabidopsis* plants [20]. *AtMBF1c* was highly induced by high salt, dehydration, heat, H_2_O_2_, methyl viologen and pathogen infection [20,21,22]. Overexpression of *CaMBF1* decreased high salt and cold stresses tolerance in *Arabidopsis* [23]. The expression level of *StMBF1* in potato stems was enhanced by heat shock and H_2_O_2_ treatments [24]. Simultaneous treatment with high temperature and drought could induce the expression of MBF1 in tobacco [21].

Chrysanthemum is a world famous kind of cut flower and is susceptible to salt stress [25]. An et al. [26] demonstrated that overexpression of *CcSOS1* improved the salt tolerance capacity in chrysanthemum. *DgNAC1*-overexpressed chrysanthemum held a higher survival rate under drought and salt stresses [27,28]; Overexpression of *DgWRKY2*, *DgWRKY4* and *DgWRKY5* genes enhanced salt tolerance in chrysanthemum [12,29,30]. To better understand the role MBF1 played in response to salt stress in chrysanthemum, we isolated a MBF1 gene from chrysanthemum and called it *DgMBF1*. Overexpression of *DgMBF1* in chrysanthemum enhanced salt tolerance in plants, indicating that the *DgMBF1* could be served as a new positive regulator of plants under salt stress.

## 2. Results

### 2.1. DgMBF1 Clone and Sequence Analysis 

A salt-responsive multiprotein bridging factor gene identified from chrysanthemum was named as *DgMBF1*. The full-length cDNA of *DgMBF1* was determined through polymerase chain reaction (PCR) and inserted into pCAMBIA 2300 controlled by the cauliflower mosaic virus (CaMV) 35S promoter. The obtained vector was transformed into the leaf disc of chrysanthemum by Agrobacterium tumefaciens. The expression level of *DgMBF1* was measured through quantitative real-time PCR (qRT-PCR). Two fully overexpressed (OE) lines (OE-3, OE-34) was selected for subsequent experiments independently.

**Figure 1:**
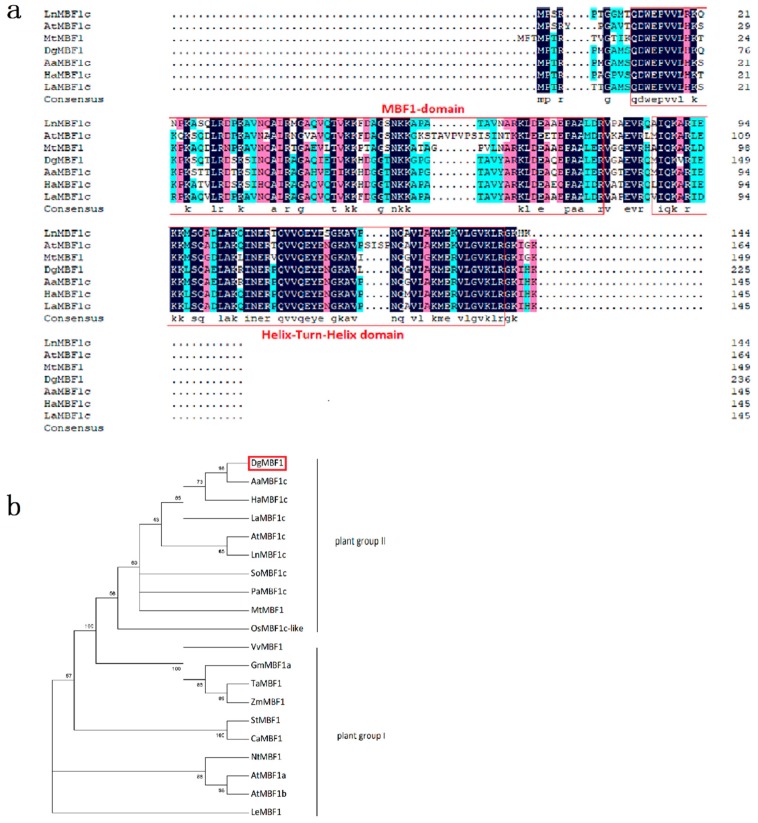
Sequence analysis of *DgMBF1*. (**a**) Multiple alignments of predicted amino acid sequences of *DgMBF1* with other plant MBF1 proteins. The shade of colors is used to distinguish the degree of consistency. Dark blue: completely consistent; pink:≥ 75%; light blue: ≥ 50%. Red frame is used to circle their domains. (**b**) Phylogenetic analysis of *DgMBF1* protein sequence with other plant MBF1 proteins. *DgMBF1* is highlight with a red frame. MBF1 proteins used in this analysis were as follows: *AaMBF1c* (*Artemisia annua*, PWA60867.1), *HaMBF1c* (*Helianthus annuus*, XP_022027306.1), *LaMBF1c* (*Lactuca sativa*, XP_023735987.1), *AtMBF1c* (*Arabidopsis thaliana*, AEE76905.1), *LnMBF1c* (*Lpomoea nil*, XP_019191938.1), *SoMBF1c* (*Spinacia oleracea*, XP_021838271.1), *PaMBF1c* (*Polytrichastrum alpinum*, AJG41867.1), *MtMBF1* (*Medicago truncatula*, AES76734.2), *OsMBF1c-like* (*Oryza sativa*, XP_015641831.1), *VvMBF1* (*Vitis vinifera*, XP_002280992.1), *GmMBF1a* (*Glycine max*, XP_003527342.1), *TaMBF1* (*Triticum aestivum*, ACO36694.1), *ZmMBF1* (*Zea mays*, ACG33346.1), *StMBF1* (*Solanum tuberosum*, AF232062.1), *CaMBF1* (*Capsicum annuum*, JX402927.1), *NtMBF1* (*Nicotiana tabacum*, BAB88859.1), *AtMBF1a* (*Arabidopsis thaliana*, AF370280), *AtMBF1b* (*Arabidopsis thaliana*, AF326909.1). *LeMBF1* (*L. esculentum*, AF096246).

The full-length *DgMBF1* gene was 733 bp, in which a 438 bp open reading frame (ORF) consisted. The ORF could encode 153 amino acids. Sequence alignments by DNAMAN showed that the *DgMBF1* protein contained an MBF1 domain at the N-terminal region and a helix-turn-helix (HTH) domain at the C-terminal region (Figure 1a). *DgMBF1* shared 92% identity with *AaMBF1c* (*Artemisia annua*, PWA60867.1), 83% with *HaMBF1c* (*Helianthus annuus*, XP_022027306.1) and *LaMBF1c* (*Lactuca sativa*, XP_023735987.1), 76% with *LnMBF1c* (*Lpomoea nil*, XP_019191938.1), 73% with *AtMBF1c* (*Arabidopsis thaliana*, AEE76905.1), and 71% identity with *MtMBF1* (*Medicago truncatula*, AES76734.2) (Figure 1b). Phylogenetic analysis indicated that *DgMBF1* was significantly more homologous to MBF1c than to MBF1a and MBF1b. Therefore, *DgMBF1* was classified as a member of the plant group II MBF1c protein.

### 2.2. Expression Analysis of DgMBF1

**Figure 2:**
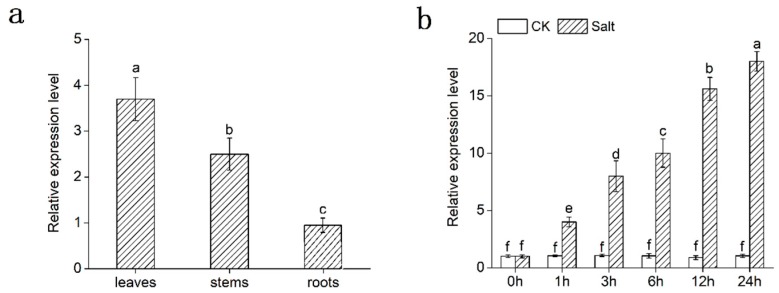
Quantitative real-time PCR analysis of *DgMBF1* expression in different tissues and in response to salt treatment. (**a**) Expression patterns of *DgMBF1* in leaves, stems and roots. (**b**) Salt treatment. Data represent means and standard errors of three replicates. Different letters above the columns indicate significant differences (*p* < 0.05) on the basis of Duncan’s multiple range test.

The expression profile of *DgMBF1* in different tissues was detected by qRT-PCR. The result suggested that the relative expression of *DgMBF1* was highest in leaves, followed by stems, and lowest in roots, and *DgMBF1* was expressed in the flowers (Figure 2a). Expression patterns of *DgMBF1* gene in leaves under salt stress were also detected by qRT-PCR. Under salinity, *DgMBF1* transcript increased continuously until 24 h and remained at a higher level compared to untreated control (Figure 2b). The result indicated that *DgMBF1* was involved in salt tolerance.

### 2.3. Observation of Callus and Phenotype 

**Figure 3:**
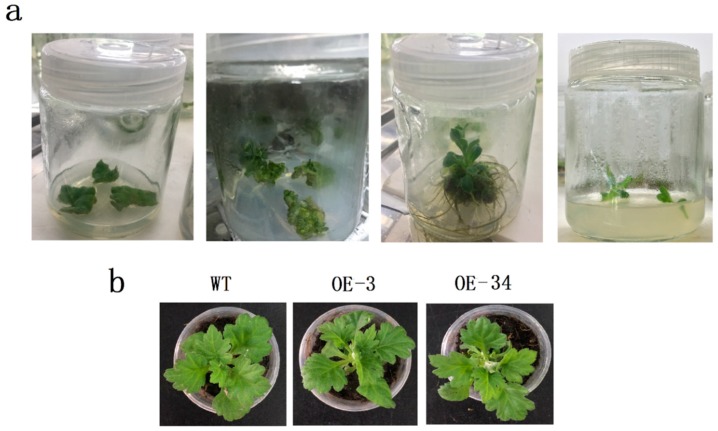
Callus transformation process and phenotypic observation. (**a**) Transgenic chrysanthemum callus transformation process. (**b**) Phenotypic observation of WT and transgenic chrysanthemums.

The growth of small buds on the infested leaf disc in chrysanthemum leaves is shown in Figure 3a. Positive plants were screened through DNA detection. Under normal conditions, there was no significant difference in phenotype between WT and transgenic chrysanthemum plants (Figure 3b).

### 2.4. Overexpression of DgMBF1 in Chrysanthemum Enhanced the Salt Tolerance

**Figure 4:**
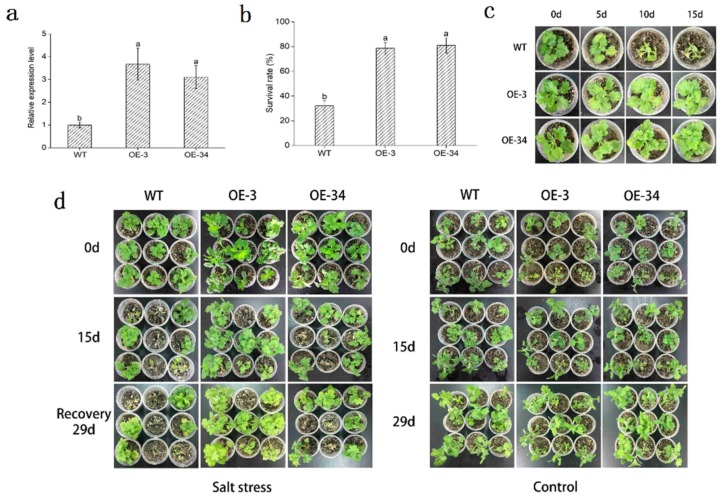
Overexpression of *DgMBF1* in transgenic chrysanthemum resulted in enhanced tolerance to salt stress. (**a**) Transcript levels of *DgMBF1* in WT and OE lines. (**b**) The survival rates of OE lines and WT after two weeks’ recovery. (**c**) Phenotypic comparison of OE lines and WT under salt stress. (**d**) OE lines and WT grown under normal (control) and salt stress conditions, followed by a recovery. Data represent means and standard errors of three replicates. The different letters above the columns indicate significant differences (*p* < 0.05) according to Duncan’s multiple range test.

To further verify the function of *DgMBF1*, we generated chrysanthemum transgenic plants. Two independent OE lines (OE-3, OE-34) were obtained and the transcript abundance of *DgMBF1* in leaves were determined by qRT-PCR (Figure 4a). The result indicated that *DgMBF1* transcript expression in two OE lines was significantly (*p* < 0.05) higher than that in WT. Under normal conditions, no obvious differences in phenotypes were observed between OE lines and WT lifelong (Figure 4d). Under salt treatment, leaves of WT plants turned yellow and even wilted with the gradual increase of salinity. While OE lines remained green and exhibited significantly (*p* < 0.05) higher survival rate than that of WT (Figure 4c,d). Moreover, the survival rates of OE-3 and OE-34 were 78.65% and 80.92%, respectively. In contrast, the WT was only 32.13% (Figure 4b).

### 2.5. Influence of Salt Stress on the Growth and Development of Chrysanthemum 

**Figure 5:**
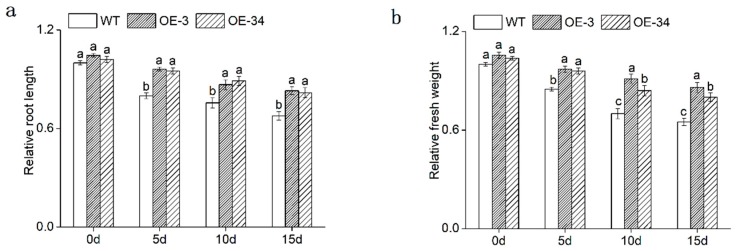
Assay of root length, fresh weight in OE lines and WT under salt stress. (**a**) Relative root length. (**b**) Relative fresh weight. Root length and fresh weight are relative to that of WT for 0 days. The different letters above the columns indicate significant differences (*p* < 0.05) according to Duncan’s multiple range test.

To study the salt impact on the growth and development of chrysanthemum, the root length and fresh weight were measured. Salinity inhibited the growth of chrysanthemum; all lines exhibited the atrophy of root and the reduction of fresh weight. While the reduction rate of OE lines was far (*p* < 0.05) smaller compared with WT (Figure 5).

### 2.6. Overexpression of DgMBF1 Enhanced Oxidation Tolerance under Salt Stress

**Figure 6:**
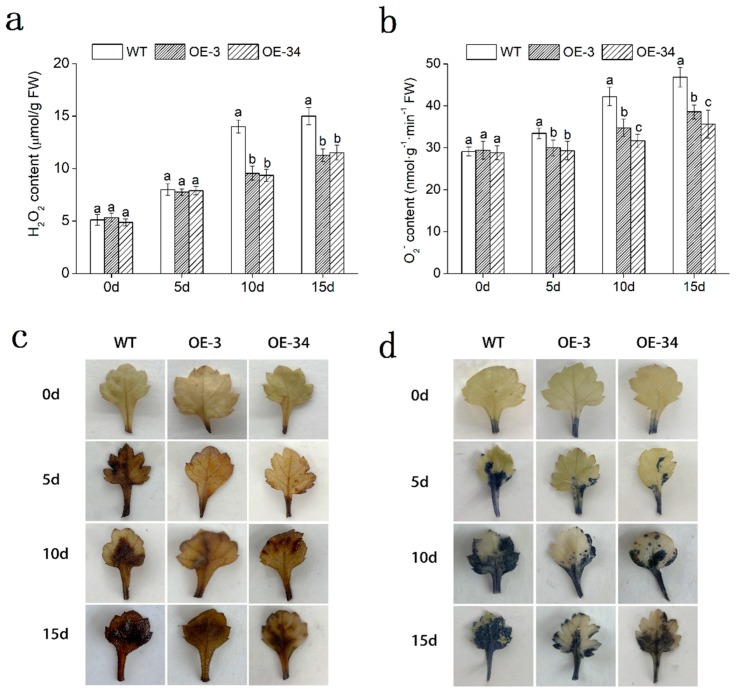
Analysis of ROS accumulation levels under salt stress. (**a**,**b**) Quantitative measurement of H_2_O_2_ and O_2_^−^ contents. (**c**,**d**) Analysis of H_2_O_2_ and O_2_^−^ contents by NBT staining and DAB staining. Data represent means and standard errors of three replicates. The different letters above the columns indicate significant differences (*p* < 0.05) according to Duncan’s multiple range test.

**Figure 7:**
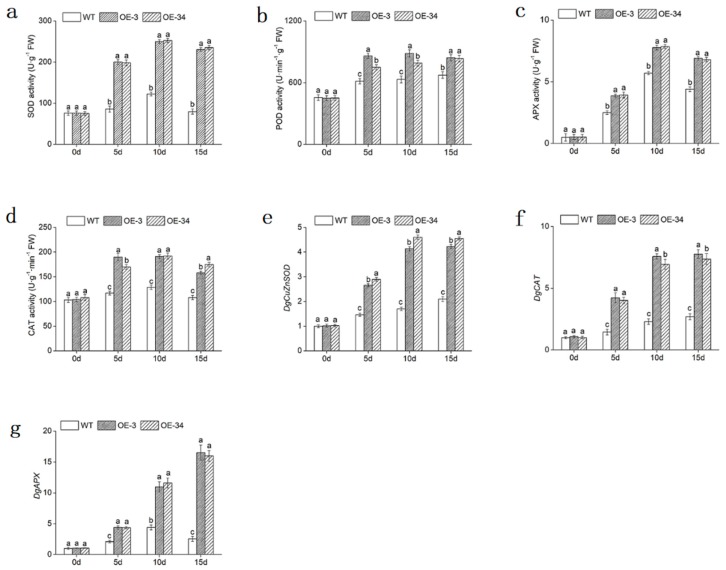
Overexpression of *DgMBF1* conferred enhanced antioxidant activities. (**a**–**d**) The SOD, POD, APX and CAT activities in WT and OE lines under salt stress. (**e**–**g**) Expression of antioxidant enzymes related genes in WT and OE lines under salt stress. Data represent means and standard errors of three replicates. The different letters above the columns indicate significant (*p* < 0.05) differences according to Duncan’s multiple range test.

The accumulation of H_2_O_2_ and O_2_^−^ in leaves was determined by diaminobenzidine (DAB) and nitro blue tetrazolium (NBT) staining in both WT and OE lines to measure the oxidation of chrysanthemum directly. The OE lines accumulated less H_2_O_2_ and O_2_^−^ than the WT with NaCl solutions treatment, for less brown and blue spots were observed (Figure 6c,d). Quantitative analysis also indicated that H_2_O_2_ and O_2_^−^ contents in leaves increased under salt stress in both WT and OE lines. OE lines (*p* < 0.05) accumulated less H_2_O_2_ and O_2_^−^ than WT. Under 15 days of salt stress, the content of H_2_O_2_ in OE lines increased to 2.11- and 2.36-fold, while the WT increased to 2.93-fold. The content of O_2_^−^ in OE lines increased by 31.08% and 23.52% than day 0, less than that in WT (Figure 6a,b). In addition, antioxidant enzymes including superoxide Anion (APX), peroxidase (POD), superoxide (SOD) and catalase (CAT) in leaves also exhibited higher activities in OE lines than that in WT plants (Figure 7a,d). Moreover, we examined the relative expression of *DgCuZnSOD*, *DgCAT* and *DgAPX*, which related to ROS-scavenging system in leaves. The results revealed that the transcript accumulation of *DgCuZnSOD*, *DgCAT*, and *DgAPX* were statistically (*p* < 0.05) higher in OE lines than that in WT under salt stress (Figure 7e,g).

Taken together, through regulating the expression of these antioxidant-related genes, overexpressed *DgMBF1* respond to against ROS persecution. Less H_2_O_2_ and O_2_^−^ contents and higher antioxidant enzyme activities would eliminate stress-induced ROS. Thus, the salt tolerance in transgenic chrysanthemum would be elevated.

### 2.7. Overexpression of DgMBF1 Promoted the Accumulation of Osmotic Substances under Salt Stress

**Figure 8:**
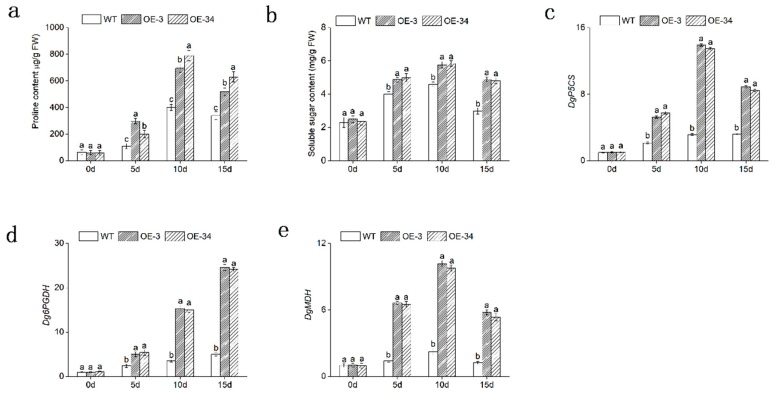
Overexpression of *DgMBF1* promotes the accumulation of osmotic substances. (**a**,**b**) The proline and SS contents in WT and OE lines under salt stress. (**c**–**e**) Relative expression level of genes involved in metabolism of proline and soluble sugars in WT and OE lines under salt stress. Data represent means and standard errors of three replicates. The different letters above the columns indicate significant (*p* < 0.05) differences according to Duncan’s multiple range test.

Proline and SS contents in leaves were measured to determine the regulation of osmotic mechanism in chrysanthemum under salt stress. The OE lines and WT exhibited little differences in the contents of proline and SS under normal conditions. However, the contents of these two osmotic regulatory factors both increased in OE lines and WT under salinity. The proline and SS highly accumulated in the OE lines (*p* < 0.05). By day 10, proline and SS contents in transgenic chrysanthemum reached a maximum; and by day 15, proline content was proximately 1.53- and 1.85-fold greater than that in WT. SS content was about 1.6- and 1.53-fold greater than that in WT. (Figure 8a,b). The relative expression of genes in leaves, related to proline biosynthesis and glycol-metabolism, including *DgP5CS*, *Dg6PGDH*, and *DgMDH* were also significantly (*p* < 0.05) up-regulated in OE lines compared with that in WT plants under salt stress (Figure 8c,e). 

These results showed that overexpression of *DgMBF1* conferred a higher osmotic pressure on transgenic chrysanthemum to cope with the dehydration stress caused by salinity. 

### 2.8. Overexpression of DgMBF1 Enhanced the K^+^/Na^+^ Selectivity under Salt Stress

**Figure 9:**
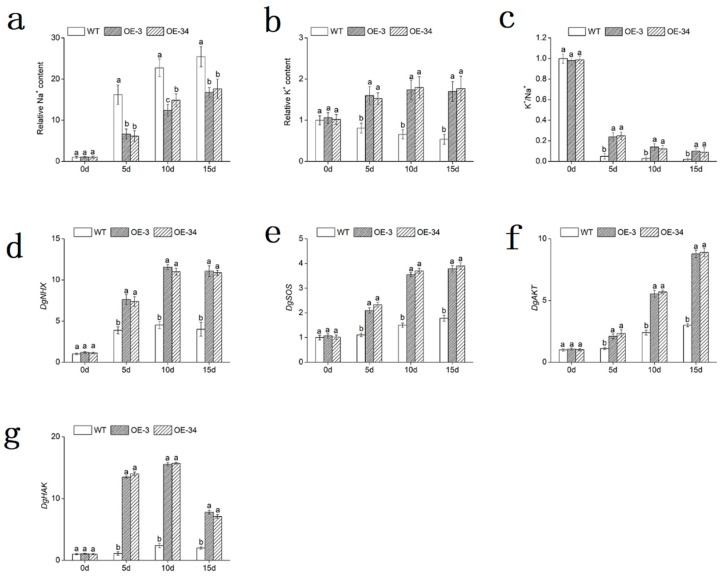
Overexpression of *DgMBF1* enhances the K^+^/Na^+^ selectivity. (**a**) Relative Na^+^ content. (**b**) Relative K^+^ contents. (**c**) K^+^/Na^+^ ratio. (**d**–**g**) Relative expression level of ion transporter genes in WT and OE lines under salt stress. Na^+^ and K^+^ contents are relative to that of WT for 0 days. Data represent means and standard errors of three replicates. The different letters above the columns indicate significant (*p* < 0.05) differences according to Duncan’s multiple range test.

The contents of K^+^ and Na^+^ in leaves were measured, without obvious differences under normal conditions. Having been exposed to salinity, the Na^+^ content of OE lines was significantly (*p* < 0.05) lower than that of WT, whereas the OE lines maintained a significantly (*p* < 0.05) higher level of K^+^ content compared to WT plants. Na^+^ content in OE lines increased approximately by 15.54- and 17.1-fold, respectively, while in WT, Na^+^ content was 25.45-fold higher than day 0. K^+^ content in OE lines revealed approximately 1.6- and 1.74-fold higher than day 0, respectively, while in WT decreased by 46%. In addition, the K^+^/Na^+^ ratio was significantly (*p* < 0.05) higher in OE lines than that in WT, which was about 4.95- and 4.46-fold greater compared with WT. (Figure 9a–c). 

Moreover, some ion transporter genes, which served to regulate the K^+^/Na^+^ homeostasis, were found in chrysanthemum leaves, including the vacuolar Na^+^/H^+^ antiporter *DgNHX*, the plasma membrane Na^+^/H^+^ antiporter *DgSOS*, the potassium channel protein *DgAKT* and the high-affinity potassium ion transporter protein *DgHAK*. The expression levels of these genes were all statistically significantly (*p* < 0.05) up-regulated in OE lines than that in WT under salt stress (Figure 9d–g).

Overall, through regulating the expression of ion transporter genes, the OE lines excluded Na^+^ and imported K^+^ more effectively than did WT in response to salt stress.

## 3. Discussion

Chrysanthemum belonged to the herbaceous perennial plants. Because of Its high medicinal and aesthetic value, the chrysanthemum-related industry thrived. However, salinity hampered the production badly [19].

Therefore, transgenic technology would be applied to improve the salinity tolerance of chrysanthemum. In this study, a multiprotein bridging factor 1 gene *DgMBF1* from chrysanthemum was isolated. The results showed that overexpression of *DgMBF1* elevated the salt tolerance of chrysanthemum.

Phylogenetic analysis of *DgMBF1* and *MBF1s* in other plant species indicated sharp differences between MBF1c (plant group II) and MBF1a/b (MBF1a and MBF1b, plant group I) proteins. However, the functional differences between the two branches have not been discovered yet. Both members were acclaimed to be involved in stress response. Our results indicated that *DgMBF1* belonged to the group II (Figure 1b) [31,32]. The transcription of *DgMBF1* in chrysanthemum leaves was dramatically promoted under salinity (Figure 2b), indicating that *DgMBF1* might participate salt stress respondence.

To further verify the function of *DgMBF1*, this gene was overexpressed in chrysanthemum. The OE lines exhibited higher salt tolerance compared with WT. Salt stress inhibited the growth and development of plants, reducing root length and decreasing fresh weight [30]. In our study, the gradually increased salinity inhibited the root length and fresh weight of chrysanthemum. The growth inhibition of OE lines was significantly (*p* < 0.05) lower than that of WT (Figure 5). These results accorded with previous studies of MBF1 genes in other plant species. For instance, overexpression of *TaMBF1c* conferred heat tolerance in rice and yeast [33]. *VvMBF1* was induced by dehydration stress and ABA treatment, and overexpression of *VvMBF1* improved drought tolerance in *Arabidopsis* [34].

Excessive ROS could cause severe damage to plants. The antioxidant system of plants would reduce cell damage caused by ROS while maintain ROS balance [35,36]. The analyses showed that the contents of H_2_O_2_ and O_2_^−^ in OE lines were lower than that in WT, and the activities of ROS scavengers (SOD, POD, APX, CAT) were statistically significant (*p* < 0.05) higher than that of WT under salt stress (Figure 6 and Figure 7a–d), which was consistent with the significant up-regulation of antioxidant related genes (*DgCuZnSOD*, *DgCAT* and *DgAPX*) (Figure 6e–g). Cu/ZnSOD was a metal enzyme that acted as a major superoxide scavenger. The overexpression of the *AhCu/ZnSOD* gene improved drought and salt tolerance in tobacco [37]. The highly activated CAT, as the plant’s cleaning agents, spared plants from the harm of reactive oxygen. It has been reported that overexpression of *GhCAT1* could raise the salt tolerance in cotton [38]. APX was an enzyme that removed H_2_O_2_ in plants. The overexpression of *TsApx6* increased the survival rate of *Arabidopsis thaliana* under drought and high salinity stress [39]. 

The results above indicated that ROS accumulation was less in *DgMBF1*-overexpressed chrysanthemum than that in WT plants under salt stress. The overexpression of *DgMBF1* activated ROS-scavenging system, increasing the salinity tolerance of chrysanthemum.

Salt stress accounted for cytoplasmic water loss, which led to osmotic stress [40]. As two important intracellular osmotic regulatory factors: proline and SS functioned well in osmotic stress response [41]. In this study, the OE lines accumulated more contents of proline and SS than that of WT plants under salt stress (Figure 8a,b). Additionally, the expression of the related genes, such as *DgP5CS*, *Dg6PGDH* and *DgMDH*, which functioned in osmotic adjustment, were statistically significant (*p* < 0.05) up-regulated in OE lines (Figure 8c–e). This was in accord with the raised contents of proline and SS. P5CS was the rate-limiting enzyme, which functioned in botanical proline biosynthesis. Overexpression of P5CS genes could increase proline production so that confer salt tolerance in transgenic plants, such as tobacco, wheat, rice and potato [42,43,44,45].

These results indicated that by regulating the osmotic adjustment ability of transgenic chrysanthemum, *DgMBF1* could improve its salt tolerance.

By regulating K^+^/Na^+^ homeostasis, *DgMBF1* conferred salt tolerance in chrysanthemum. In terms of salt tolerance, the ability to maintain a relatively high cytoplasmic K^+^/Na^+^ ratio was considered a crucial determinant [46]. Ion transporter genes played a critical role in the transport of Na^+^ in plant cells and vacuoles [47]. Salt tolerance levels were linked with the capacity to discharge Na^+^ and to maintain a relative high K^+^/Na^+^ ratio in the cells [48]. The K^+^/Na^+^ ratio exhibited little differences in OE lines and WT plants under normal conditions, while the ratio in OE lines was significantly (*p* < 0.05) higher than that in WT under salt conditions (Figure 9c). These results were supported by significant differences (*p* < 0.05) in the expression levels of certain ion transporter genes (*DgNHX*, *DgSOS*, *DgAKT* and *DgHAK*) (Figure 9a,b,d–g). In *Arabidopsis thaliana*, ion homeostasis was largely mediated by the SOS signaling pathway [49]. NHX transporters involved in cytoplasmic detoxification by vacuolar Na^+^ accumulation, osmotic adjustment by Na^+^ or K^+^ accumulation. It has been reported that *LeNHX* genes served as determinants of salt tolerance in tomato [50]. A plasma membrane Na^+^/H^+^ antiporter SOS1 influenced the export of Na^+^. The overexpression of *AtSOS1* enhanced the salt tolerance [51]. For plants growing under salt stress, it was important to maintain a Na^+^/K^+^ ratio by favoring the accumulation of potassium over sodium [52]. AKT genes encoded the root K^+^ uptake channels. The expression of a AKT1-type K^+^ channel gene *PutAKT1* enhanced salt tolerance in *Arabidopsis* [53]. K^+^ absorption and distribution in plants under salt conditions are mediated by potassium channels and transporters. The high-affinity K+ transporter (HAK) family was among the major K^+^ acquisition systems in plants [54]. Overexpression of *OsHAK5* increased the accumulation of K^+^ and improved the salinity tolerance in tobacco [55]. *McHAKs* in *Mesembryanthemum crystallinum* were significantly induced by salt stress [56].

The obvious changes in the contents of K^+^ and Na^+^, and the expression levels of *DgNHX*, *DgSOS*, *DgAKT* and *DgHAK* in the chrysanthemum together indicated that *DgMBF1* could enhance the salt tolerance of chrysanthemum by maintaining a higher K^+^/Na^+^ ratio.

At the current stage, the salt-tolerant genetic engineering technology has made major breakthrough in chrysanthemum. A variety of transgenic chrysanthemums have been proven to have better tolerance to salt stress. Due to the transgenes belonging to different families, there was no comparison with salt tolerance among various transgenic lines. High-quality genes should be the target of further study. By comparing salt tolerance to screen out the best gene, the production and cultivation of chrysanthemums would be increased. 

## 4. Materials and Methods 

### 4.1. Plant Materials and Stress Treatment

The chrysanthemum cv. ‘*Jinba*’ were potted in a 1:1 mixture of peat and perlite, and grown in an incubator with a 16h photoperiod (200 μmol·m^−2^·s^−1^ illumination), 23 ± 2 °C, 70% relative humidity. Seedlings at the 6–7 leaf stage were treated with 200 mM NaCl solutions to create salt stress. Seedlings were sampled at 0, 1, 3, 6, 12 and 24 h time points from all the treatments and stored at −80 °C for RNA extraction.

### 4.2. DgMBF1 Clone and Sequence Analyses

Total RNA was extracted from chrysanthemum leaves by the TRIzol reagent (Mylab, Beijing, China), the first-strand cDNA was synthesized with the PrimeScript™RT reagent kit (Takara, Beijing, China). A complete *DgMBF1* ORF which prepared for the inclusion of an *XbaI* and a *SacI* cloning sites was amplified from this cDNA template using the primer pair (*DgMBF1*-F/R) designed from the putative 5′ and 3′ untranslated region. The PCR products were purified by agarose gel and cloned into pMD18-T vector (Takara, Beijing, China) for sequencing.

### 4.3. Sequence Alignment and Phylogenetic Analysis

The BLAST online tool was used to search homologous protein sequences in the NCBI protein database. Sequences with over 90% coverage were selected. Sequence alignment of *DgMBF1* was performed using ClustalX. The phylogenetic tree was constructed through MEGA5.

### 4.4. Expression Vector Constructs and Chrysanthemum Transformations

Initially, the complete ORF of *DgMBF1* was cleaved by restriction with *XbaI* and *SacI* and inserted into *XbaI* and *SacI* digested the plant expression vector pCAMBIA2300 to produce pCAMBIA2300-*DgMBF1*. This pCAMBIA2300-*DgMBF1* vector was driven by the CaMV 35S promoter and transmuted into LB4404 strain of Agrobacterium tumefaciens and following the protocol documented by An et al. [57].

Middle and upper leaves of the chrysanthemum “*Jinba*” were cut into a square centimeter pieces and cultured on Murashige and skoog (MS) medium. These pieces were used as transformation receptors, pre-cultured for 3 days, the single colonies of agrobacterium LB4404 of pCAMBIA2300-*DgMBF1* were transformed into Agrobacterium liquid (cultured at 28 °C until the OD_600_ was 0.5) infecting leaf discs for 10 min, co-cultured for 3 days, delay-cultured for 3 days, the specific method according to Cui et al. [58]. Transformants were initially selected through DNA detection, chrysanthemum genomic DNA was isolated from medium-sized leaves using a DNA extraction kit (Takara, Beijing, China), primers used for DNA detection are listed in Table 1. 

### 4.5. Gene Expression Levels Analysis

The expression level of *DgMBF1* was detected by qRT-PCR using the SsoFast EvaGreen supermix (Bio-Rad, Hercules, CA, USA) and Bio-Rad CFX96TM detection system, 20 μL qRT-PCR reaction system consisted of 10μL coloring agent, 2 μL cDNA template and 300 nM primes. The settings for qRT-PCR were as follows, 95 °C 30 s, 95 °C 15 s, 60 °C 30 s, circulating for 40 times. The analyzing approach of 2^−ΔΔCt^ was applied in the experiment, using *EF1α* as the reference gene [59]. The primers used in qRT-PCR are listed in Table 1.

### 4.6. Determination of Salt Tolerance

Six transgenic strains were eventually obtained, and two independent OE lines (OE-3, OE-34) were included. The OE lines exhibited relatively high expression. These samples were reproduced through asexual propagation for salt treatment. The root, stem, leaf and flower tissues of chrysanthemum under normal condition were collected for tissue-specific expression analysis of *DgMBF1* gene.

Totally, each line contained 120 seedlings. To avoid salt shock, the method according to Chen et al. [60] was used to irrigate the chrysanthemum seedlings with an increasing concentration of NaCl solutions. Chrysanthemum seedlings at 6–7 leaves stage were irrigated with NaCl solutions: 100 mM on day 1–5, 200 mM on day 6–10, 400 mM on day 11–15, and sampled at 0, 5, 10 and 15 days.

One third of these seedlings were sampled for staining experiments, root length and fresh weight measurements. The roots were rinsed clean. The whole seedling was put into a container of known weight. The fresh weight was measured with an analytical balance. The ascending fourth and fifth leaves were cut and stained immediately. The length of 30 cm was drawn and the shallow water layer was set on the table. Stretching the wet root with the mark, the length of root was calculated. The whole root length was the sum of each sample. 

One third of seedlings were applied to statistical survival. When the salt stress treatment process was completed, the roots were rinsed with deionized water and transplanted in a new culture medium. The survival rate was counted after two-week recovery. The ascending fourth and fifth leaves collected on last one third of seedlings were used for qRT-PCR and physiological experiments.

### 4.7. Determination of Physiological Indexes of Chrysanthemum under Salt Stress

H_2_O_2_, proline and SS contents, APX, POD, SOD and CAT activities were measured with the Nanjing Jiancheng test kit, the content of O_2_^−^ was measured with the Suzhou Keming test kit. Both procedures were performed strictly following the instructions. In situ accumulation of H_2_O_2_ and O_2_^−^ were measured through histochemical staining, using DAB and NBT, respectively, following Zhao et al [28]. The Na^+^ and K^+^ concentrations were measured following An et al. [26].

### 4.8. Expression of Stress-Related Genes

Total RNA of OE lines and WT plants were isolated and converted to cDNA as described above. The expression of stress-related genes in chrysanthemum was measured by qRT-PCR. *DgCuZnSOD*, *DgCAT*, *DgAPX*, *DgP5CS*, *Dg6PGDH*, *DgMDH*, *DgNHX*, *DgSOS*, *DgAKT* and *DgHAK* were monitored, and *EF1α* as a reference. The primers used in qRT-PCR are listed in Table 1.

### 4.9. Statistical Analysis

All experiments were conducted in three biological repetitions. All data were analyzed using SPSS version 20.0 program, the significance discriminate analysis (*p* < 0.05) was performed according to Duncan’s multiple range test.

## 5. Conclusions

To conclude, our study identified a multiprotein bridging factor 1 *DgMBF1* as a salt-tolerant positive regulatory gene. *DgMBF1* was induced by salinity. The overexpressed *DgMBF1* enhanced the salt tolerance in transgenic chrysanthemum through maintaining a higher K^+^/Na^+^ ratio, greater activities of antioxidant enzymes, higher accumulation of osmotic regulatory factors. Therefore, *DgMBF1* could be served as a candidate gene for salt-tolerant plant breeding. The next step was to conduct an in-depth study of the downstream target genes of *DgMBF1* to understand its deeper molecular mechanisms in salt stress response.

## Figures and Tables

**Table 1:** Primers used in this study.

	Forward Primers	Reverse Primers
**Primers used for cloning of *DgMBF1***		
*DgMBF1*	TCCAGACCCTCAACTCCTA	ACAAACTCGACACAATACAAAG
DNA detection	GAGTCAAAGATTCAAATAGAGGACCT	ACAAACTCGACACAATACAAAG
**Primers used for qRT-PCR**		
*DgMBF1*	TGCCGACAAGACCAATGGG	TGGACAACTTGCGGCCTTT
*EF1α*	TTTTGGTATCTGGTCCTGGAG	CCATTCAAGCGACAGACTCA
*DgCuZnSOD*	CCATTGTTGACAAGCAGATTCCACTCA	ATCATCAGGATCAGCATGGACGACTAC
*DgCAT*	TACAAGCAACGCCCTTCAA	GACCTCTGTTCCCAACAGTCA
*DgAPX*	GTTGGCTGGTGTTGTTGCT	GATGGTCGTTTCCCTTAGTTG
*DgP5CS*	TTGGAGCAGAGGTTGGAAT	GCAGGTCTTTGTGGGTGTAG
*Dg6PGDH*	CGAGGTACTTCGTTCTCCGG	CTCCCTTCTTCCCCGGTAGA
*DgMDH*	GGTTGCCCCAGATGATCACA	GTTGGTCATCCAGATCGCCA
*DgNHX*	TGGTGGTAAAAGCTCGCACA	TCATTAACAACGCCCTCCCC
*DgSOS*	AGCTTCGACAAAGGGATGGG	GCTTTCTCGTCGGCTACCTT
*DgAKT*	ATTGCAGCACTTTCAGCAGC	ACTTGCCAAAGGTCCAACCA
*DgHAK*	TGGAACTTGCCATGGCCAA	GGCTTCCAACAACTGCAGC

## Abbreviations

APX	superoxide Anion
CaMV	cauliflower mosaic virus
CAT	catalase
DAB	diaminobenzidine
HTH	helix-turn-helix
H_2_O_2_	hydrogen peroxide
MBF1	multiprotein bridging factor 1
NBT	nitro blue tetrazolium
O_2_^−^	superoxide anion
OE	overexpressed
ORF	open reading frame
PCR	polymerase chain reaction
POD	peroxidase
qRT-PCR	quantitative real-time PCR
ROS	reactive oxygen species
SOD	superoxide
SS	soluble sugar
WT	wild type

## References

[1] Zhu J.K. (2002). Salt and drought stress signal transduction in plants. Annu. Rev. Plant Biol..

[2] Zhao Q., He L., Wang B., Liu Q., Pan Y., Zhang F., Jiang B., Zhang L., Liu G., Jia Y. (2018). Transcriptome Comparative Analysis of Salt Stress Responsiveness in Chrysanthemum (*Dendranthema grandiflorum*) Roots by Illumina and Single-Molecule Real-Time-Based RNA Sequencing. DNA Cell Biol..

[3] Kaleem F., Shabir G., Aslam K., Rasul S., Manzoor H., Shah M., Khan A. (2018). An overview of the genetics of plant response to salt stress: present status and the way forward. Appl. Biochem. Biotech..

[4] Munns R., Tester M. (2008). Mechanisms of Salinity Tolerance. Annu. Rev. Plant Biol..

[5] Deinlein U., Stephan A., Horie T., Luo W., Xu G., Schroeder J. (2014). Plant salt-tolerance mechanisms. Trends Plant Sci..

[6] Apel K., Hirt H. (2004). Reactive oxygen species: metabolism, oxidative stress, and signal transduction. Annu. Rev. Plant Biol..

[7] Tripathy B., Ralf O. (2012). Reactive oxygen species generation and signaling in plants. Plant Signal. Behav..

[8] Prabucki A., Serek M., Andersen A.S. (1999). Influence of salt stress on stock plant growth and cutting performance of *Chrysanthemum morifolium Ramat*. J. Hortic. Sci. Biotech..

[9] Lee M., Iersel M. (2008). Sodium chloride effects on growth, morphology, and physiology of chrysanthemum (*Chrysanthemum X morifolium*). HortScience.

[10] Lindemose S., O’Shea C., Jensen M., Skriver K. (2013). Structure, function and networks of transcription factors involved in abiotic stress responses. Int. J. Mol. Sci..

[11] Nakashima K., Ito Y., Yamaguchi-Shinozaki K. (2009). Transcriptional regulatory networks in response to abiotic stresses in *Arabidopsis* and grasses. Plant Physiol..

[12] Wang K., Wu Y., Tian X., Bai Z., Liang Q., Liu Q., Pan Y., Zhang L., Jiang B. (2017). Overexpression of *DgWRKY4* enhances salt tolerance in chrysanthemum seedlings. Front. Plant Sci..

[13] Alavilli H., Lee H., Park M., Lee B. (2017). *Antarctic moss* multiprotein bridging factor 1c overexpression in *Arabidopsis* resulted in enhanced tolerance to salt stress. Front. Plant Sci..

[14] Kim M., Lim G., Kim E., Ko C., Yang K., Jeong J., Lee M., Kim C. (2017). Abiotic and biotic stress tolerance in *Arabidopsis* overexpressing the multiprotein bridging factor 1a (MBF1a) transcriptional co-activator gene. Biochem. Biophys. Res. Commun..

[15] Mauro M., Iglesias M., Arce D., Valle E., Arnold B., Tsuda K., Yamazaki K., Casalongué C., Godoy A. (2012). MBF1s regulate ABA-dependent germination of *Arabidopsis* seeds. Plant Signal. Behav..

[16] Brendel C., Gelman L., Auwerx J. (2002). Multiprotein bridging factor-1 (MBF-1) is a cofactor for nuclear receptors that regulate lipid metabolism. Mol. Endocrinol..

[17] Takemaru K., Harashima S., Ueda H., Hirose S. (1998). Yeast co-activator MBF1 mediates GCN4-dependent transcriptional activation. Mol. Cell Biol..

[18] Fan G., Zhang K., Huang H., Zhang H., Zhao A., Chen L., Chen R., Li G., Wang Z., Lu G. (2017). Multiprotein-bridging factor 1 regulates vegetative growth, osmotic stress, and virulence in *magnaporthe oryzae*. Curr. Genet..

[19] Tsuda K., Tsuji T., Hirose S., Yamazaki K. (2004). Three *Arabidopsis* MBF1 homologs with distinct expression profiles play roles as transcriptional co-activator. Plant Cell Physiol..

[20] Tsuda K., Yamazaki K. (2004). Structure and expression analysis of three subtypes of *Arabidopsis* MBF1 genes. Biochim. Biophys. Acta.

[21] Rizhsky L., Liang H., Mittler R. (2002). The combined effect of drought stress and heat shock on gene expression in tobacco. Plant Physiol..

[22] Suzuki N., Rizhsky L., Liang H., Shuman J., Mittler R. (2005). Enhanced tolerance to environmental stress in transgenic plants expressing the transcriptional co-activator multiprotein bridging factor 1c. Plant Physiol..

[23] Guo W., Chen R., Du X., Zhang Z., Yin Y., Gong Z., Wang G. (2014). Reduced tolerance to abiotic stress in transgenic *Arabidopsis* overexpressing a *Capsicum annuum* multiprotein bridging factor 1. BMC Plant Biol..

[24] Arce D., Tonón C., Zanetti M., Godoy A., Hirose S., Casalongué C. (2006). The potato transcriptional co-activator *StMBF1* is up-regulated in response to oxidative stress and interacts with the TATA-box binding protein. J. Biochem. Mol. Biol. Biophys..

[25] Wu Y., Wang T., Wang K., Liang Q., Bai Z., Liu Q., Pan Y., Jiang B., Zhang L. (2016). Comparative analysis of the chrysanthemum leaf transcript profiling in response to salt stress. PLoS ONE.

[26] An J., Song A., Guan Z., Jiang J., Chen F., Lou W., Fang W., Liu Z., Chen S. (2014). The over-expression of *Chrysanthemum crassum CcSOS1* improves the salinity tolerance of chrysanthemum. Mol. Biol. Rep..

[27] Wang K., Zhong M., Wu Y., Bai Z., Liang Q., Liu Q., Pan Y., Zhang L., Jiang B., Jia Y. (2017). Overexpression of a chrysanthemum transcription factor gene *DgNAC1*, improves the salinity tolerance in chrysanthemum. Plant Cell Rep..

[28] Zhao Q., Zhong M., He L., Wang B., Liu Q., Pan Y., Jiang B., Zhang L. (2018). Overexpression of a chrysanthemum transcription factor gene *DgNAC1*, improves drought tolerance in chrysanthemum. Plant Cell Tissue Organ.

[29] He L., Wu Y., Zhao Q., Wang B., Liu Q., Zhang L. (2018). Chrysanthemum *DgWRKY2* gene enhances tolerance to salt stress in transgenic chrysanthemum. Int. J. Mol. Sci..

[30] Liang Q., Wu Y., Wang K., Bai Z., Liu Q., Pan Y., Zhang L., Jiang B. (2017). Chrysanthemum WRKY gene *DgWRKY5* enhances tolerance to salt stress in transgenic chrysanthemum. Sci. Rep..

[31] Suzuki N., Sejima H., Tam R., Schlauch K., Mittler R. (2011). Identification of the MBF1 heat-response regulon of *Arabidopsis thaliana*. Plant J..

[32] Zhang Y., Zhang G., Dong Y., Guo J., Huang L., Kang Z. (2009). Cloning and characterization of a MBF1 transcriptional co-activator factor in wheat induced by stripe rust pathogen. Acta Agron. Sin..

[33] Qin D., Wang F., Geng X., Zhang L., Yao Y., Ni Z., Peng H., Sun Q. (2015). Overexpression of heat stress-responsive *TaMBF1c*, a wheat (*Triticum aestivum* L.) multiprotein bridging factor, confers heat tolerance in both yeast and rice. Plant Mol. Biol..

[34] Yan Q., Hou H., Singer S., Yan X., Guo R., Wang X. (2014). Grape *VvMBF1* gene improves drought stress tolerance in transgenic *Arabidopsis thaliana*. Plant Cell Tissue Organ.

[35] Mittler R., Vanderauwera S., Gollery M., Breusegem F. (2004). Reactive oxygen gene network of plants. Trends Plant Sci..

[36] Pan Y., Wu L., Yu Z. (2006). Effect of salt and drought stress on antioxidant enzymes activities and SOD isoenzymes of liquorice (*Glycyrrhiza uralensis fisch*). Plant Growth Regul..

[37] Negi N., Shrivastava D., Sharma V., Sarin N. (2015). Overexpression of CuZnSOD from *Arachis hypogaea* alleviates salinity and drought stress in tobacco. Plant Cell Rep..

[38] Luo X., Wu J., Li Y., Nan Z., Xing G., Wang Y., Zhang A., Wang Z., Xia G., Tian Y. (2013). Synergistic effects of *GhSOD1* and *GhCAT1* overexpression in cotton chloroplasts on enhancing tolerance to methyl viologen and salt stresses. PLoS ONE.

[39] Li Z., Zhang J., Li J., Li H., Zhang G. (2016). The functional and regulatory mechanisms of the *Thellungiella Salsuginea* ascorbate peroxidase 6 (*TsAPX6*) in response to salinity and water deficit stresses. PLoS ONE.

[40] Yang Y., Shah J., Klessig D. (1997). Signal perception and transduction in plant defense responses. Genes Dev..

[41] Watanabe S., Kojima K., Ide Y., Sasaki S. (2000). Effects of saline and osmotic stress on proline and sugar accumulation in *Populus euphratica* in vitro. Plant Cell Tissue Organ.

[42] Yamchi A., Rastgar J., Mousavi A., Karkhane A., Renu (2007). Proline accumulation in transgenic tobacco as a result of expression of *Arabidopsis* 1-pyrroline-5-carboxylate synthetase (P5CS) during osmotic stress. J. Plant Biochem. Biot..

[43] Vendruscolo E., Schuster I., Pileggi M., Scapim C., Molinari H., Marur C., Vieira L. (2007). Stress-induced synthesis of proline confers tolerance to water deficit in transgenic wheat. J. Plant Physiol..

[44] Kumar V., Shriram V., Kavi K., Jawali N., Shitole M. (2010). Enhanced proline accumulation and salt stress tolerance of transgenic Indica rice by over-expressing *P5CSF129A* gene. Plant Biotechnol. Rep..

[45] Hmida-Sayari A., Gargouri-Bouzid R., Bidani A., Jaoua L., Savouré A., Jaoua S. (2005). Overexpression of Δ1-pyrroline-5-carboxylate synthetase increases proline production and confers salt tolerance in transgenic potato plants. Plant Sci..

[46] Maathuis F., Amtmann A. (1999). K^+^ nutrition and Na^+^ toxicity: the basis of cellular K^+^/Na^+^ ratios. Ann. Bot..

[47] Rus A., Estan M., Gisbert C., Garcia-Sogo B., Serrano R., Caro M. (2010). Expressing the yeast *HAL1* gene in tomato increases fruit yield and enhances K^+^/Na^+^ selectivity under salt stress. Plant Cell Environ..

[48] Yue Y., Zhang M., Zhang J., Duan L., Li Z. (2012). *SOS1*, gene overexpression increased salt tolerance in transgenic tobacco by maintaining a higher K^+^/Na^+^ ratio. J. Plant Physiol..

[49] Rodriguez-Rosales M., Galvez F.J., Huertas R., Aranda M.N., Baghour M., Cagnac O., Venema K. (2009). Plant NHX cation/proton antiporters. Plant Signal Behav..

[50] Galvez F.J., Baghour M., Hao G., Cagnac O., Rodríguez-Rosales M., Venema K. (2012). Expression of *LeNHX* isoforms in response to salt stress in salt sensitive and salt tolerant tomato species. Plant Physiol. Bioch..

[51] Yang Q., Chen Z., Zhou X., Yin H., Li X., Xin X., Hong X., Zhu J.K., Gong Z. (2009). Overexpression of SOS (Salt Overly Sensitive) genes increases salt tolerance in transgenic *Arabidopsis*. Mol. Plant.

[52] Fuchs I., Stolzle S., Ivashikina N., Hedrich R. (2005). Rice K+uptake channel *OsAKT1* is sensitive to salt stress. Planta.

[53] Ardie S.W., Liu S., Takano T. (2010). Expression of the AKT1-type K^+^ channel gene from *Puccinellia tenuiflora*, *PutAKT1*, enhances salt tolerance in *Arabidopsis*. Plant Cell Rep..

[54] Shen Y., Shen L., Shen Z., Jing W., Ge H., Zhao J., Zhang W. (2016). The potassium transporter *OsHAK21* functions in the maintenance of ion homeostasis and tolerance to salt stress in rice. Plant Cell Environ..

[55] Horie T., Sugawara M., Okada T., Taira K., Kaothien-Nakayama P., Katsuhara M., Shinmyo A., Nakayama H. (2011). Rice sodium-insensitive potassium transporter, *OsHAK5*, confers increased salt tolerance in tobacco BY2 cells. J. Biosci. Bioeng..

[56] Su H., Golldack D., Zhao C., Bohnert H.J. (2002). The expression of HAK type K^+^ transporters is regulated in response to salinity stress in common ice plant. Plant Physiol..

[57] An G., Watson B., Chiang C. (1986). Transformation of tobacco, tomato, potato, and *Arabidopsis thaliana* using a binary Ti vector system. Plant Physiol..

[58] Cui X., Chen F., Chen S. (2009). Establishment of regeneration and transformation system of ground cover chrysanthemum *Yuhuaxunzhang*. J. Nanjing Agric. Univ..

[59] Livak K., Schmittgen T. (2001). Analysis of relative gene expression data using real-time quantitative PCR and the 2^−ΔΔCt^ method. Methods.

[60] Chen L., Chen Y., Jiang J., Chen S., Chen F., Guan Z., Fang W. (2012). The constitutive expression of Chrysanthemum *dichrum ICE1* in *Chrysanthemum grandiflorum* improves the level of low temperature, salinity and drought tolerance. Plant Cell Rep..

